# Short-Term impacts of pulsed electromagnetic field therapy in middle-aged university's employees with non-specific low back pain: A pilot study

**DOI:** 10.12669/pjms.35.4.49

**Published:** 2019

**Authors:** Nermeen Mohamed Abdelhalim, Ahmed Fathy Samhan, Walid Kamal Abdelbasset

**Affiliations:** 1 Nermeen Mohamed Abdelhalim, Department of Physical Therapy and Health Rehabilitation, College of Applied Medical Sciences, Prince Sattam Bin Abdulaziz University, Alkharj City, Saudi Arabia; 2 Ahmed FathySamhan, Department of Physical Therapy and Health Rehabilitation, College of Applied Medical Sciences, Prince Sattam Bin Abdulaziz University, Alkharj City, Saudi Arabia; 3 Walid Kamal Abdelbasset Department of Physical Therapy and Health Rehabilitation, College of Applied Medical Sciences, Prince Sattam Bin Abdulaziz University, Alkharj City, Saudi Arabia

**Keywords:** Non-specific Low Back Pain, Pulsed Electromagnetic Field Therapy, Health-Related Quality of Life

## Abstract

**Objective:**

Non-specific low back pain (non-specific LBP) is common problem between office-work employees. This study aimed to evaluate the short-term impacts of Pulsed Electromagnetic Field (PEMF) therapy in the treatment of non-specific LBP symptoms as pain, back mobility, LBP disabilities, and Health–Related Quality of Life (HRQOL).

**Methods:**

Forty-two University’s employees with non-specific LBP and aged from 35 to 55 years who participated in this study from January to June 2018 were divided into two groups: group A; received PEMF therapy and group B; received sham treatment. The outcome measures were; numerical rating scale, Modified Oswestry LBP Disability Score, Modified Schober test, and the Short Form-36 questionnaire. Evaluations were performed for both groups before and after finishing treatment.

**Results:**

All outcome measures were significantly improved statistically in the experimental group at the end of the intervention (p<0.05). On the other hand, there were non-significant differences in all outcome measures in the sham group (p>0.05).

**Conclusions:**

PEMT therapy may decrease pain, LBP disability, increase lumbar spine mobility, and improve HRQOL in middle-aged university’s employees with nonspecific LBP.

## INTRODUCTION

Low back pain (LBP) is one of the most common problems and it has regular causes of disability. Intercontinental, LBP usually leads to loss of functional, psychosocial affect activities of daily life (ADL) and quality of life (QoL).^1^ LBP can be categorized as mechanical, non-mechanical, and psychological. Mechanical LPB may be specific or nonspecific. Under its continuance, LBP may be acute (lower than six weeks), sub-acute (continuingsix to 12 weeks), chronic (continuingmore than 12 weeks), and recurring.^2^

Non-specific LBP is not assignable to familiar cause and corresponds 90–95% of the subjects of LBP. Nonspecific LBP is identified by no structural damages; it can restrict ADL and workperformance.^3^ The mechanism of LBP is unclear and is identified as non-specific LBP. It has a complexetiology with distinctfeatures (age, physical activity), psychosocial aspects (tension, anxiety, sadness) and workconstituents (hardmanual work, bending motions, shaking) implied in its progress.^4^

Office-work employees have been determined to have an elevated frequency and suffered from non-specific LBP,^5^ because of sitting for long time and specific bad back postures, in addition to other workplace environmental constituents,^6^ and the continuous computer practice.^7^ Health-related quality of life (HRQOL) assessment may be utilized to distinguish among patients with non-specific LBPbefore and after particular treatment method,anticipateprospectiveresults or circumstances, and assessdifferences over time.^8^

Different methods of treatments are available for non-specific LBP, such as drugs (non-steroidal anti-inflammatory, corticosteroids, muscle relaxants, etc.), physical therapy modalities (short waves diathermy, ultrasound waves, etc.), acupuncture, and injection therapy.^9^

Pulsed electromagnetic field (PEMF) therapy is established a slow frequency electromagnetic currents, with an extended range of frequencies, which will increase cell membrane permeability and stimulation of many intracellular functions.^10^ Nowadays, PEMF therapy has several advantages in treatment of different clinical manifestations as relief of pain, accelerate wound healing, edema resolution and inflammation therapy, and osteoarthritis.^11^

However, the widespread use of PEMF therapy in LBP is increasing and there are important studies on this modality, a rationalization of its impacts on the non-specific LBP is still inadequate especially in evaluation of its short-term effects in HRQOL. Therefore, this study aimed to evaluatethe short-term impacts of PEMF therapy in producing significant variability in non-specific LBP symptoms as pain &back mobility and secondary LBP disabilities & HRQOL.

## METHODS

This trial was a pilot experimentalstudy. The trial continued from January to June 2018, participants were referred from the orthopedist of Prince Sattam Bin Abdulaziz University Hospital to the outpatient physical therapy clinic (male & female sections) in the department of physical therapy and health rehabilitation, College of Applied Medical Sciences.

Forty-two University’s employees, with age ranged from 35 to 55 years, were enrolled in this study based on the following inclusion criteria; suffering from non-specific LBP diagnosed by the orthopedist, with or without radiated pain, with pain intensity more than five on the numerical rating scale (NRS), with Body Mass Index (BMI) below 30 (kg/m²), not have any pain therapy such as physical therapy interventions, injection, ornon-steroidal anti-inflammatory drugs in the last threemonthsbefore starting the study, andduring the study procedures, and had a pain lastingfor a minimum ofsix months before starting the study. Patients with cauda equina manifestations, neoplasms, metabolic disorders, rheumatoid arthritis, osteoporosis, utilizing corticosteroids for an extended period of time, nerve root compression, lumbar fixation, spinal operation, pregnancy, and cardiac pacemakerwere excluded from the study.

The pursued study protocol was in agreement with the Institutional Ethical Committee, and informed consent was signed by all participants.Patients were allocated into two groups, group A; received PEMF therapy and group B; sham treatment. CR-3000 system (CR Technology Co., Kyungki-do, Korea), which produces a PEMF therapy with low-frequency electromagnetic field with a greatest output intensity of two tesla (± 5%) and the frequency ranged from one to 50 Hz. The magnetic pulsation created is characterized by two phases and has a pulse-duration modulation of 270μs (± 5%).^12^

### Group A

(the experimental group) which consisted of 21 non-specific LBP patients were treated by PEMF therapy. Patients were laid on a bed in a comfortable and well-supported position, and then the transducer part of electromagnetic field was located five cm apart from the skin of lower back and electromagnetic pulse-duration modulation changing everyfive sec, frequencies of five to ten Hz. During each treatment session, the intensity began minimum degree and increased progressively until the patient tolerance. Finally, after finishing, the lower back was examined for any adverse effects like reddening, itching, or discomfort.

### Group B

(the sham group), 21 non-specific LBP patients were laid on the bed, the device as an exactly alike procedure of the experimental group, butthe plug of the electromagnetic cable was disconnected from the generatorand maintainedunder the device to benot watched by the patient, at the same time the patient heard the similar rhythmic voice of the device. Both groups received the treatment procedures at a rate of three sessions per week for 0ne month (12 sessions). Evaluations were performed for both groups before starting the treatment procedures, and after finishing the last session (after one month). Evaluations included the following:

***NRS:*** was an 11-degreescale where zerorevealed no pain and tendescribedsever pain.^13^

***Modified Oswestry LBP Disability Score (M-OSW):*** consists of ten parameters including; pain severity, social life, self-care, weight lift, walk, sit, stand, sleep, travel, employing/home performance.^14^ Each domain scores was ranged from zero to five and the total scores rangedfrom zero to fifty. The level of disability was intensified when total score increased.

***Modified Schober test:*** was applied to evaluate the flexion/extension range of motion (ROM) of lumbar spine.Each patient was in standing position, the reference line for lumber ROM was the junction of dents of venus on the lower back. Using a marker, a sign was represented ten cm over and five cm under the reference, the patient was informed to lean forward and backward and assessment was performed utilizing a tape measurement in mm.^15^

***HRQOL:*** was assessed utilizing the Short Form-36 questionnaire (SF-36) which included 36 parameters in form of eight domains involving; general health, intellectual health, physical ability, physical role, emotion role, social role, pain, and healthiness.^16^ Total scores were ranged from zero to100. The HRQOL wasconsidered the best with high scores and the worst with low scores.

The sample size was estimated using the primary outcome measure of the present pilot study; changes in pain severity using NRS. Adopting the effect size of 0.8 for NRS with α=0.05 and 80% power, the study required 17 patients in each group. Hence, 21 participants were included in each group to account for dropout rate of 20%.

The descriptive statistical analysis was performed using SPSS version 20.0. Means and standard deviations were demonstrated for all examined values. For assessing the normality of data distribution, Kolmogorov-Smirnov-test was carried out. Inferential statistics evaluated changes of the normally distributed data using paired and unpaired t-test, whereas the Wilcoxon signed-rank and Mann Whitney-U tests were performed to assess non-normally distributed data. *P*<0.05 was considered statistically significant.

## RESULTS

Forty-eight University’s employees with non-specific LBP were assessed for eligibility in this study. Forty-two were enrolled in the study, twenty-one in each group. Four patients did not conform to the inclusion criteria and two patients discontinued.

**Fig. 1:**
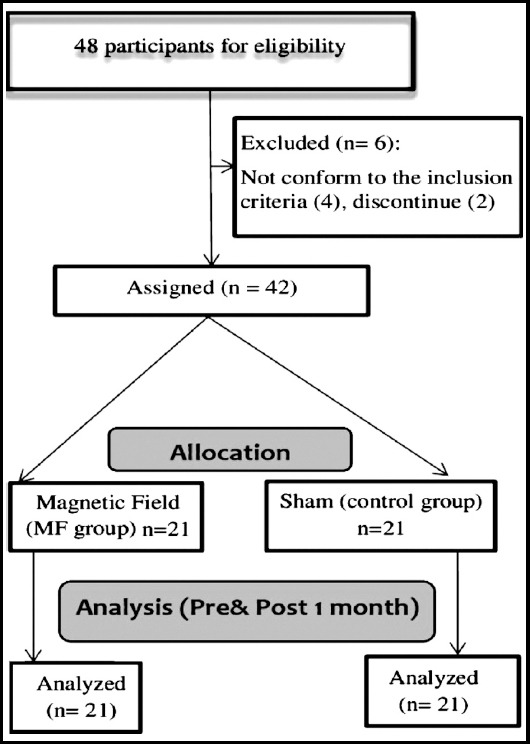
Study flow chart.

As shown in Table-I, there were no significant differences between the experimental and the sham groups in terms of baseline characteristics (p>0.05). The main findings of this study showed that all measures, including the reduction in NRS, decrease in M-OSW, increase in ROM, and improvement of HRQOL scores, were significantly improved statistically in the experimental group at the end of the intervention (p<0.05), as presented in Table-II. On the other hand, there were non-significant differences in all measurements in the sham group as p>0.05.

**Table I:** Baseline characteristics of the patients in the two groups.

Measures	Experimental group(n=21)	Sham group (n=21)	P-value
Sex (Men/Women)	15/6	13/8	0.921
Age (years)	37.03±6.74	36.85±7.14	0.933
BMI (kg/m2)	26.23±3.15	27.14±2.82	0.323
Manifestations duration (months)	23.18±7.18	22.09±8.21	0.649
NRS	6.8±2.1	6.6±2.4	0.775
M-OSW (%)	42.8±7.17	41.6±6.31	0.567
Flexion (mm)	42.3±9.8	43.6±11.2	0.691
Extension (mm)	16.2±4.5	16.9±5.1	0.639
HRQOL (total scores)	46.7±5.6	47.12±4.8	0.795

**Table II:** Changes of the outcome measures at the end of the study in the two groups.

Measures	Experimental group (n=21)			Sham group (n=21)		

	Before	After	p-value	Before	After	p-value
NRS	6.8±2.1	3.8±1.3	<0.001	6.6±2.4	6.7±2.3	0.891
M-OSW (%)	42.8±7.17	22.9±4.61	<0.001	41.6±6.31	42.2±9.4	0.809
Flexion (mm)	42.3± 9.8	54.6±11.8	<0.001	43.6±11.2	42.7±10.9	0.793
Extension (mm)	16.2±4.5	24.8±4.3	<0.001	16.9±5.1	16.5±6.3	0.822
HRQOL (total scores)	46.7±5.6	55.3±8.4	0.004	47.12±4.8	46.9±4.5	0.879

The comparison of post-treatment measures between the experimental and sham groups revealed significant differences in all outcome measures in favor of the experimental group (p<0.05) as presented in Table-III.

**Table III:** Comparison of the mean differences between the two study groups post-treatment.

Measures	Experimental group(n=21)	Sham group (n=21)	P-value
NRS	3.8±1.3	6.7±2.3	<0.001
M-OSW (%)	22.9±4.61	42.2±9.4	<0.001
Flexion (mm)	54.6±11.8	42.7±10.9	0.002
Extension (mm)	24.8±4.3	16.5±6.3	<0.001
HRQOL (total scores)	55.3±8.4	46.9±4.5	0.002

## DISCUSSION

In this study, PEMT therapy was establish to decrease pain, increase back mobility (flexion & extension), reduce LBP disability and improve HRQOL on middle-aged university’s employees with non-specific LBP. The use of pilot study design accepted the validity of the study, although a powerful sham effect was noticed, a greater improvement was regularly established in the PEMT therapy group compared with the sham group at the end of the study procedures. Patients in the sham group were advised to be treated with PEMF therapy after finishing this study to get benefits. Also, there were no critical adverse or side effects after the application of PEMF therapy.

Several recent studies have recommended that the application of PEFT alone have a wonderful impact in relieving pain in LPB patients.^12^,^17^ However, when applied in conjunction with other traditional therapies like physical therapy^18^ or analgesics^19^ appears to do not supplement a further impact to the traditional therapies.

The mechanisms of PEMF therapy in decreasing pain and inflammation are unclear and there are many theories discuss them. One theory is that PEMF therapy may produce Eddy currents in the biological tissues. Another one is gate control theory as induced by the application of electrical stimulation which may be inhibit pain signals to some extent by clear alteration of the nervous system or may be by motivation of inhibitory sensory neurons,^20^ and/or the indirect impacts of gene aspect by local interference of the electrochemical changes.^21^ Recent theory proposes that PEMF therapy can perform alteration in the gene aspect the comprising genes of pain courses like endogenous opioids and eicosanoid enzyme courses.^22^ Any of these may be submitted the underlying mechanisms liable for the outcomes of this study.

Some small studies have suggested that psychosocial stress, work for a long time, and bending forward of low back in the workplace might be professional risk factors for non-specific LBP.^5^ The importance to treat university’s employees who had non-specific LBP was of great value to improve their performance at the same time to facilitate functional activities.

More than one outcome measures was carried out to support the findings of this study. Additional trials using different modalities (continuous or interrupted), pulse widths and times of PEMT therapy as well as various follow-up times may assist to settle the ideal procedure for application of PEMF therapy.

In conclusion, the PEMT therapy is a safe, non-invasive, and applicable modality. Clinically, it may decrease pain and increase lumbar spine mobility and seems to be an efficient treatment for the improvements of LBP disability and HRQOL on middle-aged university employees with nonspecific LBP.
